# Pin site cover: a simpleton’s approach

**DOI:** 10.1308/003588412X13171221591259m

**Published:** 2012-05

**Authors:** D Mahadeva, N Bali, H Prem

**Affiliations:** Birmingham Children’s Hospital NHS Foundation TrustUK

Pin site complications after orthopaedic procedures include skin indentation and wire migration.^1^ A simple technique can be incorporated into daily practice to offset the above. After the wire is fixed and the wire tip is bent, a Primapore® dressing (Smith & Nephew, Hull, UK) is cut in the middle and secured around the pin to prevent skin indentation. To further augment this, a second Primapore® is crimped and adhered over the tip to prevent the wire getting caught in the padding and migrating. This method is cheap, readily available and easy to apply. We have avoided the above complications in a series of 57 cases.

**Figure 1:**
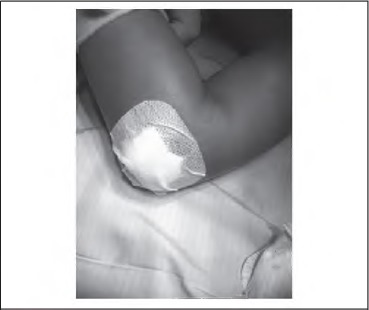
Primapore® dressing secured around pin

**Figure 2:**
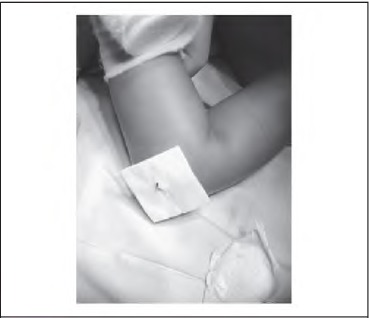
Second Primapore® dressing adhered over tip
